# Pharmacological Activities and Chemical Stability of Natural and Enzymatically Acylated Anthocyanins: A Comparative Review

**DOI:** 10.3390/ph16050638

**Published:** 2023-04-23

**Authors:** Jimena Yañez-Apam, Astrid Domínguez-Uscanga, Azucena Herrera-González, Jonhatan Contreras, Luis Mojica, Gail Mahady, Diego A. Luna-Vital

**Affiliations:** 1 Tecnologico de Monterrey, School of Engineering and Science, Ave., 2501, Monterrey 64849, Mexico; jya2396@hotmail.com (J.Y.-A.); astrid.dominguez@tec.mx (A.D.-U.); 2 Tecnologico de Monterrey, The Institute for Obesity Research, Ave., 2501, Monterrey 64849, Mexico; 3 Department of Chemical Engineering, Centro Universitario de Ciencias Exactas e Ingenierías, Universidad de Guadalajara, Blvd., Gral., Marcelino García Barragán 1421, Guadalajara 44430, Mexico; mariaa.herrera@academicos.udg.mx; 4 Food Technology, Centro de Investigación y Asistencia en Tecnología y Diseño del Estado de Jalisco A.C.-Unidad Zapopan, Camino Arenero 1227, Zapopan 45019, Mexico; jocontreras_al@ciatej.edu.mx (J.C.); lmojica@ciatej.mx (L.M.); 5 Clinical Pharmacognosy Laboratory, Department of Pharmacy Practice, College of Pharmacy, PAHO/WHO Collaborating Centre for Traditional Medicine, University of Illinois at Chicago, 833 South Wood St., Chicago, IL 60612, USA; mahady@uic.edu

**Keywords:** enzymatic acylation, polyphenols, natural pigments, pigment stability

## Abstract

Anthocyanins (ANCs) are naturally occurring water-soluble pigments responsible for conferring red, blue, and purple colors to fruits, vegetables, flowers, and grains. Due to their chemical structure, they are highly susceptible to degradation by external factors, such as pH, light, temperature, and oxygen. Naturally acylated anthocyanins have proven to be more stable in response to external factors and exhibit superior biological effects as compared with their non-acylated analogues. Therefore, synthetic acylation represents a viable alternative to make the application of these compounds more suitable for use. Enzyme-mediated synthetic acylation produces derivatives that are highly similar to those obtained through the natural acylation process, with the main difference between these two pathways being the catalytic site of the enzymes involved in the synthesis; acyltransferases catalyze natural acylation, while lipases catalyze synthetic acylation. In both cases, their active sites perform the addition of carbon chains to the hydroxyl groups of anthocyanin glycosyl moieties. Currently, there is no comparative information regarding natural and enzymatically acylated anthocyanins. In this sense, the aim of this review is to compare natural and enzyme-mediated synthetic acylated anthocyanins in terms of chemical stability and pharmacological activity with a focus on inflammation and diabetes.

## 1. Introduction

ANCs are one of the most abundant groups of naturally occurring phenolic compounds present in many flowers, fruits, vegetables, and grains [1]. In terms of their chemical structure, ANCs are water-soluble glycosylated or acyl-glycosylated forms of the flavylium cation (2-phenylbenzopyrilium) that differ in the methoxyl and/or hydroxyl substitutions on the A- and B-rings [2]. These compounds are considered pigments because they are responsible for the red, blue, and purple colors in different plant tissues [3]. Methoxylation of the chromophore, compared with hydroxylation, gives slightly redder hues. In the presence of an acid, ANCs form a stable red-colored flavylium, and under basic conditions, they partially transition to a blue quinonoid structure [3,4]. To form ANCs, their precursors, anthocyanidins or aglycones, bind with monosaccharides or oligosaccharides, such as glucose, rhamnose, galactose, or arabinose at positions C3, C5, C7, C3′, and C5′ through glycosidic bonds, with C3 being the most frequent binding point [4,5]. There are more than 500 types of ANCs in nature, of which cyanidin-3-glucoside, delphinidin-3-glucoside, pelargonidin-3-glucoside, malvidin-3-glucoside, petunidin-3-glucoside, and peonidin-3-glucoside are the most common [3,6]. ANC acylation is the process by which the hydroxyl groups (-OHs) of ANC glycosyls are partially or totally esterified by various organic acids [7]. These esterified groups are more stable than hydroxyl radicals, and the aliphatic long-chains from different organic acids, used as acylating agents, improve ANC stability [8]. Literature supports the acylation of ANCs considerably improving their overall stability and bioactivities [9]; thus, many efforts have been made to imitate this natural process in the laboratory, such as synthetic acylation. To date, only lipases have been used for synthetic acylation, mainly lipase B from *Candida antarctica*. However, due to the potential of synthetic acylation, the use of different types of enzymes should be explored. Although it is known that the synthetic acylation of ANCs results in improved stability and bioactivity, there are still several aspects that need to be explored, such as different acylation methods, novel ANC sources, and industrial applications. Since there is no comparative study of natural and enzyme-mediated synthetic acylation processes reported in the literature, this review aims to explore and discuss the effects of both types of acylation in ANCs regarding their stability and bioactivity with a focus on inflammation and diabetes. Since there are a few reports available on this topic, all relevant manuscripts were included regardless of the publication year.

## 2. Presence of Acylated Anthocyanins in Nature

**Figure 1:**
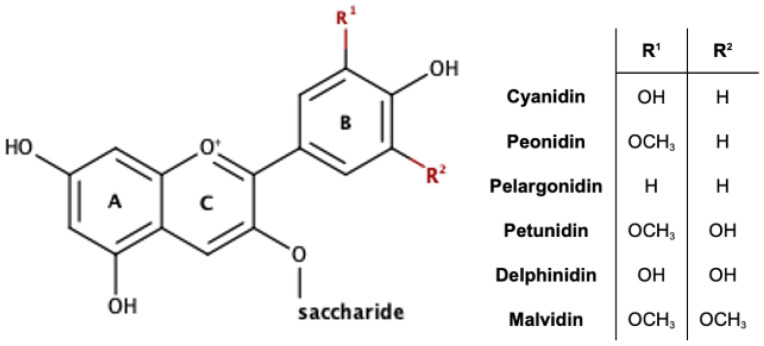
Basic chemical structure of the most common anthocyanin-3-monosaccharides.

**Figure 2:**
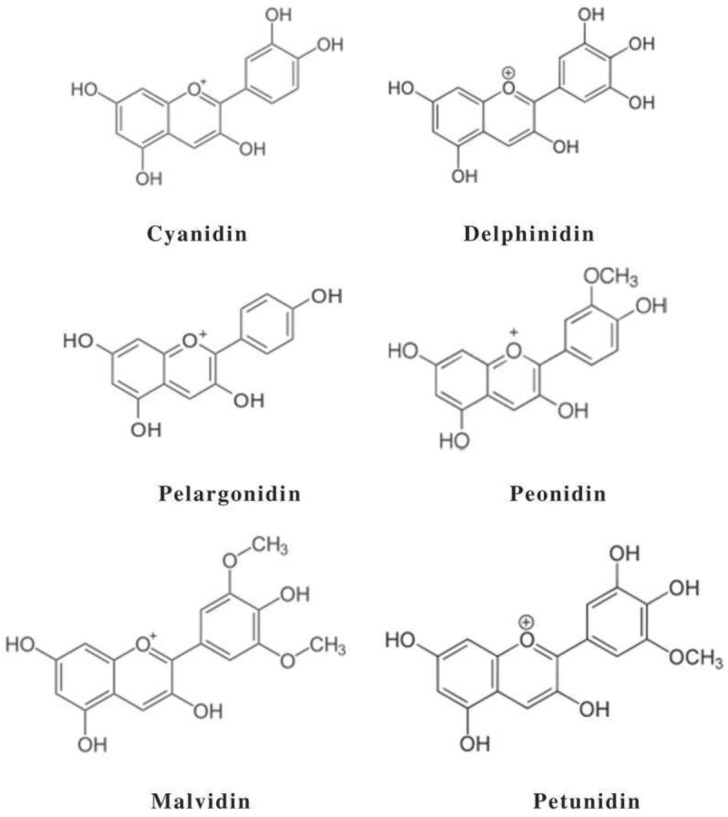
Major anthocyanidins in nature (obtained from Pubchem). Available online: https://pubchem.ncbi.nlm.nih.gov (accessed on 5 September 2022).

Chemically, ANCs are glycosylated or acyl-glycosylated forms of anthocyanidins that differ in the methoxyl and/or hydroxyl substitutions on A- and B-rings (Figure 1) [2]. Although more than twenty naturally occurring anthocyanidins have been identified, cyanidin, delphinidin, pelargonidin, peonidin, malvidin, and petunidin (Figure 2) are the most commonly reported [10]. Glycosyl acylation is one of the key aspects that provides diversity within ANC molecules and the types, numbers, and linkage positions of the acyl groups considerably increase the types of ANCs in nature [7].

The ANC biosynthetic pathway is an extension of the general flavonoid pathway, which starts with the following:

1. The synthesis of naringenin chalcone from 4-coumaroyl-CoA and malonyl-CoA mediated by chalcone synthase (CHS).
2. Then, naringenin chalcone is isomerized by chalcone isomerase (CHI) to naringenin.
3. The naringenin is converted into dihydrokaempferol by flavanone 3-hydroxylase (F3H). This compound can be further hydroxylated by flavonoid 3′-hydroxylase (F3′H) or flavonoid 3′,5′-hydroxylase (F3′5′H) into two other dihydroflavonols, dihydroquercetin or dihydromyricetin, respectively.
4. Next, the three dihydroflavonols are converted into colorless leucoanthocyanidins by dihydroflavonol 4-reductase (DFR) and subsequently to colored anthocyanidins by anthocyanidin synthase (ANS).
5. Then, sugar molecules are attached to anthocyanidins by various glycosyltransferases, for instance, flavonoid 3-O-glucosyltransferase (UFGT), yielding ANCs.

**Figure 3:**
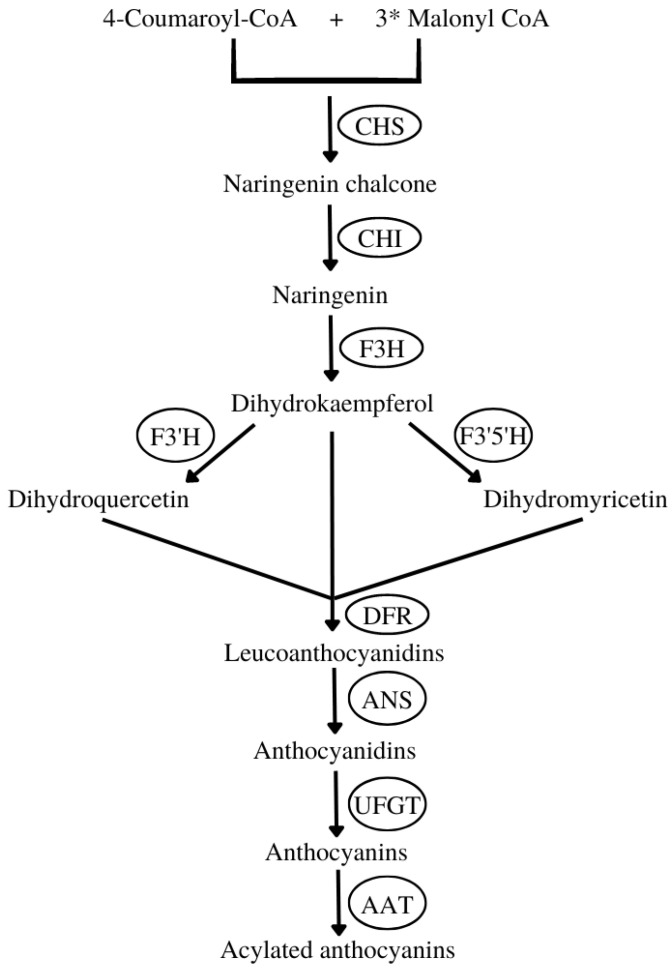
Anthocyanin biosynthetic pathway (adapted from [11]). CHS (chalcone synthase), CHI (chalcone isomerase), F3H (flavanone 3-hydroxylase), F3′H (flavonoid 3′-hydroxylase), F3′5′H (flavonoid 3′,5′-hydroxylase), DFR (dihydroflavonol 4-reductase), ANS (anthocyanidin synthase), UFGT (flavonoid 3-O-glucosyltransferase), FLS (flavonol synthase), AAT (anthocyanin acyltransferase). “*” means multiplication.

Finally, as a post-biosynthetic modification, ANCs can be further acylated with aromatic or aliphatic acyl groups by different acyltransferases (AATs) (Figure 3) [11].

Acyltransferases are classified into two distinct categories based on their acyl-donor specificity: aliphatic AATs and aromatic AATs. Aliphatic AATs act on acyl-CoA donors, such as malonyl-CoA, acetyl-CoA, methyl malonyl-CoA, and succinyl-CoA, whereas the aromatic AATs act on *p*-coumaroyl-CoA and caffeoyl-CoA. Although these enzymes are active in the cytoplasm [12], acylation can also occur in the vacuole, mediated by acyl-glucose-dependent AATs [13].

As the substitution of hydroxyl groups in the B-ring increases, a shift of the maximum wavelength of absorption (λ_max_) is obtained at longer wavelengths (bathochromic shift) resulting in a bluer hue. The sugar residues may be acylated with aromatic acids, such as *p*-coumaric, caffeic, ferulic, sinapic, gallic, or *p*-hydroxybenzoic acids, and/or aliphatic acids, such as malonic, acetic, malic, succinic or oxalic acids [14]. These acyl substituents are commonly bound to the C3 sugar, esterified to the 6-OH, or less frequently to the 4-OH group of the sugars. However, there are ANCs with very complex acylation patterns attached to different sugar moieties [15].

Some of the most common sources of natural acylated ANCs are strawberries, radishes, and red potatoes. From these sources of pigments, several ANCs have been isolated, such as raphanusin acylated with *p*-coumaric acid: pelargonidin-3-[2-glucosyl-6-trans-*p*-coumaroyl-glucoside]-5-glucoside; raphanusin + ferulic acid: pelargonidin-3-[2-glucosyl-6-trans-feruloyl-glucoside]-5-glucoside; raphanusin + *p*-coumaric and malonic acid: pelargonidin-3-[2-glucosyl-6-trans-*p*-coumaroyl-glucoside]-5-(6-malonyl-glucoside); and raphanusin + ferulic and malonic acid: pelargonidin-3-[2-glucosyl-6-trans-feruloyl-glucoside]-5-(6-malonyl-glucoside); among others. Red grapes, black carrots, purple sweet potatoes, and red cabbage are other edible sources with a significant content of this stable form of ANCs [14]. Red cabbage has been reported to be rich in acylated ANCs, mainly in diacylated forms. It contains about 15 ANC species being derivatives of cyanidin-3-diglucoside-5-glucoside acylated with ferulic, sinapic, and/or *p*-coumaric acids. Black carrots owe their color to the presence of five main ANCs being derivatives of cyanidin-3-rutinoside-glucoside-galactoside acylated with one cinnamic acid (*p*-coumaric, ferulic, or sinapic) [16].

Red radish contains a total of 12 ANCs, with eight of them diacylated, mainly pelargonidin derivatives [17]. Acylated ANCs from broccoli sprouts were identified and isolated for the first time by [18]. These ANCs were naturally acylated with one or more cinnamic acids: cyanidin 3-O-(sinapoyl) diglucoside-5-O-glucoside, cyanidin 3-O-(feruloyl) diglucoside-5-O-glucoside, cyanidin 3-O-(*p*-coumaroyl) diglucoside-5-O-glucoside, cyanidin 3-O-(*p*-coumaroyl) (sinapoyl)diglucoside-5-O-glucoside, and cyanidin 3-O-(sinapoyl) (feruloyl) diglucoside-5-O-glucoside, among others.

In purple sweet potato, 12 ANCs were identified, seven cyanidin derivatives and five pelargonidin derivatives, of which 11 compounds were acylated. The three main ANCs were peonidin 3-caffeoyl-*p*-hydroxybenzoyl sophoroside-5-glucoside, peonidin 3-(6′′-caffeoyl-6′′′feruloyl sophoroside)-5-glucoside, peonidin 3-caffeoyl sophoroside-5-glucoside, cyanidin 3-caffeoyl-*p*-hydroxybenzoyl sophoroside-5-glucoside, and cyanidin 3-(6′′-caffeoyl-6′′-feruloylsophoroside)-5-glucoside [9,19].

## 3. Effect of Natural Acylation on Anthocyanin Stability

**Figure 4:**
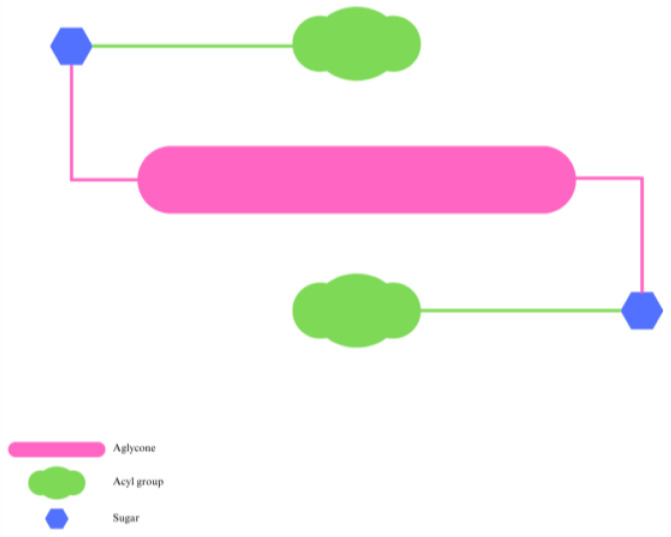
Anthocyanin intramolecular co-pigmentation.

ANCs own their intense color to their chromophore of eight conjugated double bonds carrying a positive charge on the heterocyclic oxygen ring, which makes them able to absorb light in the visible light region. The acyl groups stack with the pyrylium ring of the flavylium cation reducing susceptibility to the nucleophile attack of water and subsequent formation of a pseudobase or a chalcone (intramolecular co-pigmentation) (Figure 4) [17,20,21].

It has been reported that naturally acylated ANCs present in various flowering species are more stable compared with their non-acylated counterparts, most likely due to increased intramolecular stacking [22]. The general physical effects of acylated ANCs are decreased polarity, increased molecular size, and a modified spatial structure [23]. Other aspects that confer acylated ANCs advantages are their stability and lipotropic resistance [8]. Research involving the development of ANC-containing food colorants has led to the discovery of ANC molecules with complex patterns of glycosylation and acylation that exhibit increased stability with pH changes, heat treatment, and light exposure [14]. Acylated ANCs show less color fading with an increased pH than their non-acylated analogs [17].

Andean red sweet potato and purple corn extracts were evaluated under different external conditions and compared to commercial colorants. Overall, it was observed that red sweet potato extract and purple carrot commercial colorant, both rich in acylated ANCs, were more chemically stable at a pH range of 0.9–4 after 138 days at 20 °C as compared with purple corn extract and red grape commercial colorant, containing mainly non-acylated ANCs. Moreover, when exposing extracts and commercial colorants to 98 °C for 2 h the red sweet potato extract and purple carrot commercial colorant exhibited higher stability than the purple corn extract and red grape commercial colorant. Red sweet potato extracts maintained a red-violet hue for extended periods of time and showed comparable chemical stability to the commercial purple carrot colorant [24].

Matsufuji et al. (2007) [25] investigated the stability of red radish extract in response to light, heat, and hydrogen peroxide at different pH values. The major ANCs present in red radish extract were pelargonidin glycosides acylated with *p*-coumaric, ferulic, or caffeic acids. The results showed that diacylated ANCs are more stable in response to light (fluorescent light, 5000 lx), heat (90–95 °C), and H_2_O_2_ than monoacylated ANCs. The authors suggested that this effect is probably due to a sandwich type stacking between the planar aromatic residues of intramolecular acyl units and the flavylium nucleus. In addition, it was observed that ANCs acylated with *p*-coumaric or ferulic acids were more stable than ones acylated with caffeic acids.

Purple sweet potato is another source rich in acylated ANCs, mainly peonidin and cyanidin glycosides acylated with *p*-hydroxybenzoic, ferulic, and caffeic acids. The results presented by Chen et al. (2019) [26] showed that after 15 days of storage at 4 °C, only 20% of the ANC content of purple sweet potato decreased and the color change was acceptable. Storage at 4 °C and 25 °C, regardless of light or dark conditions, did not result in a significant color change and the decrease in the ANC content was less than 5%.

Another report on acylated ANC stability was published by Tang & Giusti (2018) [27]. Black goji berry contains approximately 80% acylated ANCs, and the following were identified: delphinidin-3-*p*-coumaroyl-rutinoside-5-glucoside (*cis* and *trans* isomers) and petunidin-3-*trans*-*p*-coumaroyl-rutinoside-5-glucoside, being the last the major pigment. After a saponification reaction, petunidin-3-rutinoside-5-glucoside was yielded to be compared with the acylated *trans* isomer petunidin-3-*trans*-*p*-coumaroyl-rutinoside-5-glucoside. Regarding color degradation, petunidin-3-*trans*-*p*-coumaroyl-rutinoside-5-glucoside suffered less color loss when the pH increased from acidic to neutral and under alkaline conditions (3–9). Furthermore, the isolated petunidin-3-*trans*-*p*-coumaroyl-rutinoside-5-glucoside showed remarkable color stability under alkaline conditions, since its purple-blue hue endured for up to three weeks.

Hu et al. (2014) [28] provided evidence of the superior stability of acylated ANCs from *Lycium ruthenicum* Murray and *Nitraria Tangutorum* Bobr. In solutions with pH 5 and 7, petunidin-3-O-rutinoside (trans-p-coumaroyl)-5-glucoside was reduced by 50% after 12 days of observation compared with its non-acylated counterpart, petunidin-3,5-O-diglucoside, which was reduced by 85%. Moreover, the photostability of acylated and non-acylated ANCs was also evaluated, and pelargonidin-3-O-(trans-coumaroyl)-diglucoside was the most stable species in light and dark environments (pH 3) reducing its ANC content by only by 10% after 10 days of light exposure. 

Lakshan et al. (2019) [29] reported the development of a natural beverage incorporating blue pea flower (*Clitoria ternatea* L.) extract. The beverage was shelf-stable at room temperature for 28 days without the addition of preservatives. The increased stability was attributed to the presence of polyacylated ANCs known as ternatins, mainly derivatives of delphinidin 3,3′,5′-triglucoside. Optimum color stability was reached at a pH range of 3.5–4, at which the extract exhibited an intense blue color.

## 4. Enzymatic Synthesis of Acylated Anthocyanins and Effect on Their Stability

Acylation is the addition of an acyl group (RCO) via electrophilic substitution, and this reaction can be accomplished using high temperatures; nevertheless, ANCs are heat-labile. Interestingly, the condensation between sugars and α-hydroxycarboxylic acids can be performed in water at 60 °C being regioselective for the primary hydroxyl groups of mannose, galactose, and glucose. Although a catalyst-free reaction is ideal, it is also difficult to achieve since some special conditions are necessary, and employable reactants are somewhat limited. In this sense, more efforts are being concentrated on developing new methods focused on green chemistry [30].

There is significant evidence supporting naturally acylated ANCs being more chemically stable than their non-acylated counterparts [4,9,19,31,32]. In this regard, acylation has played a key role in improving the overall stability of ANCs against certain external stimuli to make their use in food products more suitable. In that sense, the synthetic acylation of ANCs has gained the interest of the scientific community. Table 1 summarizes some of the most common ANC sources, reaction reagents, and main effects of synthetic ANC acylation. For example, in their study, Zhang et al., (2021) [33] described the synthesis of cyanidin-3-glucoside (C3G) conjugates with methyl fatty acids. The results of this study showed that acylated derivatives of C3G with medium-chain fatty acids had superior stability and optical absorbance than C3G. Acylation has also played an important role in improving the solubility and pH resistance of ANC. In the work by Yang et al. (2020) [34], the enzymatic synthesis of cyanidin-3-glucoside lauryl ester (C3G-C12) was compared with native C3G, and showed improved liposolubility, pH resistivity, and thermostability. In cases where the addition of non-acylated ANCs to high-fat products is not suitable due to their poor fat solubility, acylation represents a viable alternative to improve this problem. Cruz et al. (2018) [35] carried out an interesting study where they performed the enzymatic acylation of a mixture of ANCs extracted from waste blackcurrant fruit skins with octanoic acid. The ANC extract was composed of rutinosides and glucosides of cyanidin and delphinidin. The results obtained showed a selective and preferential enzymatic acylation of cyanidin and delphinidin glucosides, whereas the rutinosides remained non-acylated. The acylated ANCs showed improved general stability. Fernandez-Aulis et al. (2020) [36] developed a strategy to synthesize more stable ANCs from corn and other plant materials. In this work, they performed the enzymatic esterification of cyanidin-3-glucoside, cyanidin-3-rutinoside, and cyanidin-3,5-diglucoside using vinyl cinnamate as an acyl donor. A relevant aspect of this study is that underutilized sources that are not commonly used for ANC extraction, such as tiliapo (*Sideroxylon palmeri*), plum (*Prunus domestica*), trueno fruit (*Ligustrum japonicum*), and bottlebrush flower (*Callistemon citrinus*), where investigated. The esterified derivatives were evaluated at 40, 60, and 85 °C to assess their thermostability. All esterified products showed improved thermostability compared with C3G. The antioxidant activity was also measured by evaluating the radical-scavenging activities of the esterified products. All esterified derivatives had higher radical-scavenging activity in comparison with C3G.

The study by Liu et al., (2020) [46] described the acylation of blueberry ANCs using *p*-coumaric acid and caffeic acid as acylating agents to improve ANC color stability and antioxidant activities. The DPPH-radical-scavenging activity and inhibition ratio in the β-carotene-bleaching assay of the ANCs improved after acylation with *p*-coumaric acid and caffeic acid because of the good antioxidant properties of both acyl donors due to the presence of phenolic hydroxyl groups in their structure. The DPPH-radical-scavenging rate of the native ANCs increased by 6.56% and 15.21% after acylation with *p*-coumaric acid and caffeic acid, respectively. Likewise, the inhibition ratio in the β-carotene-bleaching assay of the native ANCs increased by 7.93% and 16.86% after grafting with the two acyl donors. In terms of color, the fading degrees of native and acylated ANCs were measured and calculated during storage at 25, 40, and 60 °C to assess the effects of acylation. The colors of the acylated derivatives remained relatively stable at 25 °C and 40 °C, and the fading degree slightly changed. At 60 °C, the fading values of the acylated derivatives rose rapidly; nevertheless, the fading values were always lower compared with the native blueberry ANCs.

Marquez-Rodriguez et al. (2021) [47] isolated delphinidin 3-O-sambubioside (Dp3sam) from roselle flower (*Hibiscus sabdariffa* L.) and esterified it with octanoic acid using *Candida antarctica* lipase B. The results showed that Dp3sam-C8 exhibited two remarkable improvements, first, stabilization of the quinoidal base at neutral or moderate alkaline pH that promoted the blue color to remain, and secondly, a higher hydration resistance compared with the native ANCs.

Marathe et al. (2021) [38] esterified red rose petal ANCs and added them to food systems. The acylation reaction was performed by the enzyme Fermase CALB^TM^ 10,000, and the acyl donor was lauric acid. The antioxidant activity of esterified ANCs was assessed and remained stable. The lauric acid esters of ANCs were applied as colorants in food products, such as cupcakes, sandwich biscuit cream filling, and puffed rice extrudates. In the case of cupcakes containing ANC lauric acid esters, they displayed an aesthetically better and more appealing color (lemon green), in comparison to the color of cupcakes, with non-esterified ANCs (moss green). For the biscuit cream, the incorporation of ANCs and their esters gave a brown hue upon mixing with the cream. The thermo-oxidative stability was studied in the presence of esterified and non-esterified ANCs, resulting in better thermo-oxidative stability of samples with ANC lauric esters compared with samples with native ANCs and a control without ANCs (blank cream). Finally, the color stability of ANC lauric esters was assessed during rice extrusion processing. Compared with native ANCs and synthetic color, the total color difference, as well as the difference in chroma, was the least for extrudates containing ANC lauric acid esters. Moreover, the total color difference during a storage period of 4 weeks showed a similar result with a significantly low difference in color.

Teng et al. (2022) [37] performed the enzymatic acylation of C3G from raspberry with methyl salicylate under reduced pressure, and the acylated derivative cyanidin-3-(6-salicyloyl) glucoside showed significantly improved stability in light, heat, and oxidative environments. The antioxidant capacity of cyanidin-3-(6-salicyloyl) glucoside showed no significant changes in DPPH, ABTS-free-radical-scavenging ability, and oxygen-free radical-absorption capacity compared with non-acylated counterparts, indicating that acylation does not affect the initial antioxidant capacity of ANCs.

In the study carried out by Zhang et al. (2021) [33], they investigated the effect of acylation on C3G lipophilicity, stability, and antioxidant capacity. C3G was enzymatically acylated through transesterification with fatty acid esters, and three monoacylated C3G esters were produced, cyanidin-3-(6′′-n-octanoyl)-glucoside, cyanidin-3-(6′′-lauroyl)-glucoside, and cyanidin-3-(6′′-myristoyl)-glucoside. Results showed that increasing the fatty acyl chain length of the derivatives led to an improvement in lipophilicity. In terms of thermostability and photostability, cyanidin-3-(6′′-n-octanoyl)-glucoside had the best results compared with the other C3G esters.

In our recent study [48], a complex phenolic-rich extract from purple corn (PCE) was enzymatically treated with lipase B in acetonitrile using octanoic acid as an acyl donor. Acylated and non-acylated PCE were tested for thermal stability at 80 °C and ɑ-glucosidase-inhibitory activity. The enzymatic treatment promoted the generation of a reaction product identified via HPLC, and this increased the half-life of total ANCs in solution at 80 °C. Additionally, the lipase treatment enhanced the ɑ-glucosidase-inhibitory activity at the highest concentration of mg eq. C3G/mL.

By analyzing the works focused on the synthetic acylation of ANCs, even though natural acylation is mediated by AATs and synthetic acylation is mediated by lipases, it can be observed that the enzymatic method shares key characteristics with the acylation process that occurs in nature, such as the site and position where the acylation occurs, as well as the improvements in the overall stability of ANC molecules. In that sense, Table 2 summarizes the similarities and differences between natural and synthetic ANC acylation processes.

### Enzymes Used for Anthocyanin Acylation

Enzymes play an important role in acylation technology. Enzymatic acylation may be accelerated by the continuous azeotropic removal of water, using adsorptive control with the aid of molecular sieves. One key step during acylation reactions is the separation of the enzyme once the reaction ends, and the immobilization of enzymes leads to easy separation, higher enantioselectivity, catalytic activity, and storage stability. Lipases are widely used for converting carboxylic acids and alcohols into esters in organic solvents in a highly stereoselective manner when a chiral acid is employed. This technique is advantageous because enzymes are more stable in organic solvents than in water, and some substrates and products are unstable in aqueous solution as well. Chitosan immobilizes microbial lipases from *Candida antarctica* B to allow for repeated uses in the acylation of carboxylic acids with various alcohols. Silica gels are also popular supports for enzyme immobilization [30].

Lipases (E.C. 3.1.1.3) catalyze the hydrolysis of ester bonds between alcohols and carboxylic acids. Their preferred substrates are triglycerides with long-chain fatty acids that are insoluble in water. In terms of structure, lipases have two main conformations: a closed and an open conformation. The main difference between both conformations is that in the closed form, the amphiphilic α-helix, commonly named lid, isolates the active center from the medium, whereas in the open one, the lid is displaced, and the hydrophobic residues are exposed around the active site [51].

Two notable features make these enzymes advantageous for a wide range of applications; they have good specificity against a vast range of substrates and besides, present a high enantio- and regioselectivity. *Candida antarctica* lipase B (CalB), recently reclassified as *Pseudozyma antarctica* lipase B (PalB), is a member of the α/β-hydrolase fold family with a Ser-His-Asp/Glu catalytic triad. It contains two mobile α-helices surrounding the active site (α5 and α10) that contribute to the ability of the enzyme to bind many different substrates. As many lipases are stable in organic solvents, they are suitable for catalyzing ester bond formation or transesterification reactions [51].

**Figure 5:**
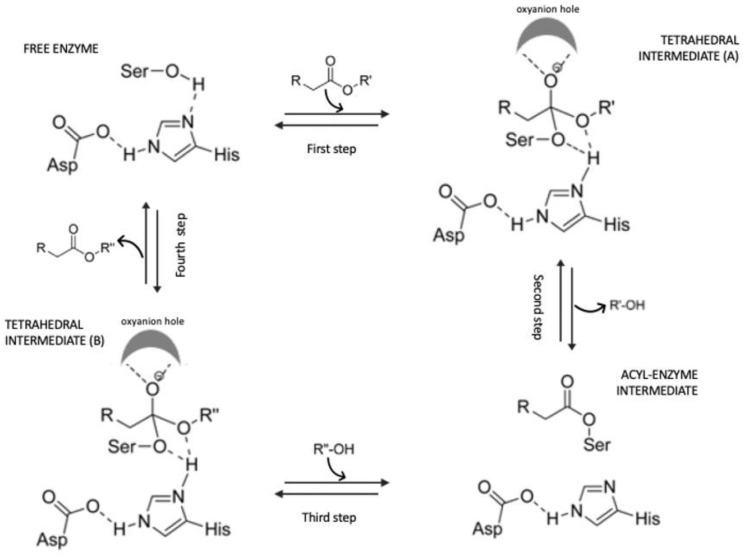
The catalytic mechanism of lipases (adapted from [53]).

The catalytic mechanism can be explained as follows: firstly, when an ester substrate binds to the active site, the nucleophilic hydroxyl group of the catalytic serine attacks the carbonyl carbon atom of the ester. Thus, a tetrahedral intermediate (A) is generated and the negative charge is stabilized by hydrogen bonding by threonine and glutamine residues that are located at the oxyanion hole. Then, the alcohol is eliminated, and the acyl-enzyme intermediate is generated. Later, the attack of the second substrate (alcohol) on the carbonyl carbon atom of the acyl-enzyme intermediate results in the formation of the second tetrahedral intermediate (B). Finally, the collapse of the second tetrahedral intermediate (B) produces an ester that is released, and the active site of the enzyme is regenerated [52,53] (Figure 5).

**Figure 6:**
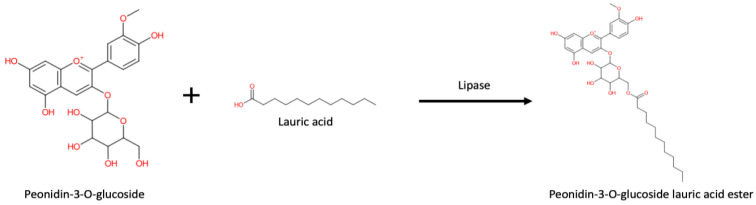
A possible graft of lauric acid on peonidin-3-O-glucoside generated using IBM RXN for Chemistry (https://rxn.res.ibm.com accessed on 12 August 2022).

It has been widely reported that the acylation of many ANCs takes place at the primary alcohol C6″-OH of the glucose or galactose moiety [54]. As an example of enzymatic acylation, the result of a simulation generated using the IBM RXN for Chemistry software using peonidin-3-O-glucoside (P3G) and lauric acid is shown (Figure 6).

## 5. Relevance of Natural Acylated and Non-Acylated Anthocyanins in Inflammation and Diabetes

Table 3 summarizes research describing different in vitro, in vivo, and clinical studies that evaluated the effects of naturally acylated ANCs in obesity and diabetic models. In an in vitro study, Zhang et al. (2019) [55] investigated the associations between the phenolic composition of purple maize ANC-rich water (PMW) extract and its anti-inflammatory, anti-adipogenic, and anti-diabetic properties. A total of seven ANCs were identified, including three glycosides (C3G, Pr3G, P3G), three acylated glycosides (C3G-Mal, Pr3G-Mal, P3G-Mal), and a flavanol–ANC dimer (catechin-cyanidin-3,5-diglucoside). The results showed that C3G, P3G, and their acylated forms were the main constituents responsible for the biological activities of PMW. The results further showed that the PMW extract reduced the secretion levels of the inflammatory markers TNF-α and IL-6. Moreover, the extract inhibited the activity of α-amylase, which may be beneficial for patients with T2DM by controlling the postprandial blood glucose excursion.

Another in vitro study performed by Cai et al. (2020) [57] on the effect of acylated ANCs on inflammation induced by hypoxia and ischemia was performed using cultured SH-SY5Y neuroblastoma cells. The acylated ANC petunidin-3-O-rutinoside (*p*-coumaroyl)-5-O-glucoside was previously purified from the dried fruits of *Lycium ruthenicum* Murray for its use. Oxygen and glucose deprivation (OGD)-treated SH-SY5Y cells were treated with different concentrations of ANCs (10 μg/mL, 100 μg/mL, and 1000 μg/mL). OGD-treated cells with no ANCs showed reduced viability, while OGD-treated cells with ANCs showed that ANCs significantly increased the cell viability, with the optimal concentration being 100 μg/mL. In a concentration-dependent study, ANC treatment of the cells at 50 μg/mL, 100 μg/mL, and 200 μg/mL showed that ANCs increased cell viability in a concentration-dependent manner. Using these concentrations, cells were protected with ANCs, and after 30 min of OGD, the levels of TNF-α, IL-1β, and IL-6 in the supernatant of SH-SY5Y cells were detected. The results showed that ANCs significantly reduced the elevated levels of these markers. Yang et al. (2021) [60] compared the inhibition activities of acylated and non-acylated ANC fractions from blueberries and purple sweet potatoes against α-amylase and α-glucosidase. Briefly, for the in vitro analysis, α-amylase and α-glucosidase, separately, were mixed with different ANC fractions and then incubated. Acarbose was used as the positive control, and phosphate-buffered saline (PBS) solution without samples was the blank control. The absorbance of the solution was recorded at 405 nm, and the results were the following: diacylated AF-PSP (ANCs from purple sweet potato) showed the best inhibitory effects on α-amylase (IC_50_ = 0.078 mg mL^−1^) among all ANC fractions, but it was weaker than that of acarbose (IC_50_ = 0.0048 mg mL^−1^). Moreover, diacylated AF-PSP was found to suppress α-glucosidase activity (IC_50_ = 1.56 mg mL^−1^) more effectively than the other fractions, showing a similar result to acarbose (IC_50_ = 1.66 mg mL^−1^). The ANC fractions from blueberries were only moderate inhibitors of α-glucosidase; however, monoacylated AF-PSP showed no effect against these enzymes. Additionally, the effects of diacylated AF-PSP on starch digestion in vivo were assessed. Male Sprague Dawley (SD) rats were randomly divided into three groups: normal control group (control), which did not receive ANC administration; low-dose diacylated AF-PSP group with a dose of 80 mg kg^−1^; and high-dose diacylated AF-PSP group with a dose of 160 mg kg^−1^. Subsequently, cooked starch was administered intragastrically to all groups. Blood samples were collected up to 120 min just after the administration of starch, and then, blood glucose levels were determined using a glucometer, and the area under the curve (AUC) values over 120 min were calculated. The results show that both treatments of 80 mg kg^−1^ and 160 mg kg^−1^ diacylated AF-PSP significantly decreased the postprandial blood glucose levels after 30 min of starch administration compared with those in the control group. Blood glucose levels exhibited about 10.11% and 20.52% reductions for the 80 mg kg^−1^ and 160 mg kg^−1^ diacylated AF-PSP groups, respectively. In another in vivo study performed by Wu et al. (2018) [63], C57BL/6 mice were randomly divided into groups and fed two different types of diets, a high-fat-diet and low-fat-diet with the addition of blackberries or blueberries in their daily food for 12 weeks. The main ANC in blackberry is cyanidin-3-glucoside, and for blueberry, the main ANCs are cyanidin-3-glucoside and cyanidin-3-rutinoside. The expression levels of inflammatory markers, such as IL-6, TNF-α, and NF-κB, in liver tissue were measured. The results showed that the administration of blackberries and blueberries downregulated gene expression levels of IL-6, TNF-α, and NF-κB when compared with those observed in mice fed a high-fat diet only. Moreover, blueberries were more effective in reducing inflammatory markers than blackberries.

Bell et al. (2017) [64] evaluated the impact of ANC-rich wild blueberries on the postprandial glucose response in a small clinical trial. Seventeen healthy young adults consumed different doses of ANC-rich wild blueberry powder, at 0 mg, 310 mg, and 724 mg, in both sugar-matched and no-added-sugar conditions. After drink ingestion, blood glucose levels were measured at pre-consumption and 150 min postprandially. The outcomes of this study showed that the highest ANC dose (724 mg) extended the glycemic response with blood glucose remaining significantly elevated at 120 min as compared with baseline levels, and the lowest ANC dose (310 mg) exhibited similar results at 90 min postprandially. The consumption of ANC-rich wild blueberry powder during the study also showed an interesting metabolic benefit, preventing reactive hypoglycemic episodes where the blood glucose level fell close to or below normal levels. Zhang et al. (2020) [65] performed a 12-week, randomized, double-blind, placebo-controlled clinical trial involving 169 eligible subjects with dyslipidemia. The subjects were supplemented with multiple doses of ANCs, and the oxidative and inflammatory responses were evaluated. Supplementation was provided in the form of oral capsules (Medox^®^) that contained ANC extracts from wild Norwegian bilberries and blackcurrants that contained 17 different natural ANCs purified from both berries. Subjects were randomly assigned to one of the four dosing groups proposed: placebo, ANCs at 40, 80, and 320 mg/day. The absolute changes in inflammatory cytokine concentrations from baseline to follow-up (at 6 and 12 weeks) were assessed within the groups. The results of the study showed that after 6 weeks, the levels of inflammatory cytokines did not significantly change, and after 12 weeks, 40 mg/day ANCs moderately reduced the serum IL-6 and TNF-α from the baseline, but the results were not statistically significant. However, the 12-week ANC supplementation at a dose of 80 mg/day or 320 mg/day resulted in a significant decrease in serum IL-6 and TNF-α. In a recent clinical trial performed by Nikbakht et al. (2021) [66], the anti-inflammatory effect of ANCs in type 2 diabetic (T2D), T2D-at-risk, and healthy individuals was evaluated. Participants were assigned to 4 weeks of ANC intervention in capsule form (Medox^®^) at a daily dose of 320 mg ANCs (four capsules of 80 mg), extracted from bilberries and blackcurrants. Pre- and post-intervention samples were screened for pro-inflammatory biomarkers, including interleukin-8 (IL-8), interleukin-18 (IL-18), interleukin-1R alpha (IL-1Rα), tumor necrosis factor-alpha (TNF-α), resistin, and leptin. The results showed a statistically significant reduction in IL-6, IL-18, and TNF-α in diabetic participants; however, IL-1Rα, IL-8, and leptin did not differ in any of the groups.

Jokioja et al. (2020) [59] performed a clinical trial to investigate the effect of yellow-fleshed potatoes, with and without the addition of purple potato extract (PPE) rich in acylated ANCs, on glycemia and insulinemia in the postprandial state in healthy men. The results showed that, for blood glucose, the incremental area under the curve at 120 min was significantly lower for the meal containing PPE as compared to that with the control meal without the PPE extract. Moreover, the meal with the PPE extract caused a statistically significantly lower postprandial glucose response at 20 min and 40 min as compared with the control meal. The PPE-containing study meal also reduced the steep increase in the levels of plasma glucose and insulin as compared with the control meal. In an in vivo study performed by Chen et al. (2020) [58], male Zucker Diabetic Fatty (ZDF) rats were fed acylated ANCs extracted from purple potatoes (AAPP) and non-acylated ANCs extracted from bilberries (NAAB) to evaluate the metabolic impact of these compounds on the plasma metabolic profile of diabetic rats. Both acylated and non-acylated ANCs resulted in lower levels of blood glucose. Additionally, the group fed AAPP showed a significant decrease in lactate, citrate, and pyruvate, indicating improved glycolysis. Moreover, the AAPP from purple potatoes decreased the levels of lactate, serine, and glycine, which was associated with an improved oxidative status in diabetic rats. Along with other fruits and vegetables, red cabbage is an important source of naturally acylated ANCs. Buko et al. (2018) [56] evaluated the protective effect of a red cabbage extract (RCE) in rats with streptozotocin-induced diabetes. The results of this study showed that rats fed RCE had lowered blood glucose concentrations. The antihyperglycemic effect of the extract was confirmed based on the final fasting glucose concentration, which was significantly lower in the blood of rats treated with RCE as compared to that in the untreated group. Interestingly, the treatment with RCE increased serum insulin, pro-insulin, and C-peptide concentrations in STZ-induced diabetic rats suggesting the activation of insulin synthesis.

## 6. Green and Sustainable Alternatives for Enzymatic Acylation

Currently, there is one report on the use of green solvents and sustainable alternatives for enzymatic acylation. As a green alternative to common organic solvents, ionic liquids (ILs) have been widely used lately for many chemical reactions and even biotransformation [67]. Ionic liquids are organic salts possessing unique physicochemical properties, including low toxicity, reduced vapor pressure, non-volatility, higher thermal stability, and high solvation properties [68].

In an enzymatic reaction, when there is a suitable combination of cations and anions in the medium, the solubility of the substrate increases; thus, the enzyme selectivity improves, and its activity remains stable. Therefore, ILs seem to be a viable alternative for enzymatic ANC synthesis [67]. Recent trends in microwave (MW)-assisted synthesis have used ILs as solvents, co-solvents, and/or catalysts since their ionic nature allows for the very effective coupling with microwave energy [69]. MW synthesis has been demonstrated to contribute to enzyme stabilization within the reaction due to its conformational change, allowing the substrate to reach the active site of the enzyme more easily under MW irradiation [67].

Guimarães et al. (2020) [67] attempted, for the first time, the production of a cyanidin-3-glucoside-octanoic acid conjugate combining ionic liquids (ILs), microwave (MW) irradiation, and a *Candida Antarctica* lipase B as a biocatalyst. They concluded that using [BMIm][OTf] (1-butyl-3-methylimidazolium trifluoromethanesulfonate) instead of 2M2B as a solvent could considerably reduce the reaction time allowing for the formation of acylated products with similar yields to the conventional method. The highlight of their work is the importance of achieving the perfect cation and anion compositions for the ILs to generate the desired enzymatic reaction. These promising results represent a starting point to continue producing this type of ANC derivatives under more environmentally friendly conditions.

## 7. Perspectives and Conclusions

Acylation reactions can improve the stability and biological potential of ANCs. This work focused on comparing synthetic and natural acylation processes. Recent studies showed that enzyme-mediated synthetic acylation produces highly similar derivatives compared to the natural pathway, with the main difference between synthetic and naturally occurring synthesis being the enzyme catalytic site involved in the reaction. Acyltransferases catalyze natural acylation, while lipases catalyze synthetic acylation; in both reactions, the addition of carbon chains to the hydroxyl groups of ANC glycosyl sites takes place at the active site of the enzymes. However, the synthetic acylation reaction has several challenges and opportunities to be addressed, including improving the reaction conditions by generating enzyme mutants with higher efficiency for synthesizing acylated ANCs. Moreover, the reaction conditions, quantification, and characterization of final products can be optimized by using novel experimental designs. Regarding an environmentally friendly approach, the use of green solvents in the acylation reactions represents an interesting alternative for obtaining food-grade derivatives that can be safely applied in industry. On the other hand, the successful application of naturally occurring acylated ANCs and synthetic acylated ANCs in the food industry requires an evaluation of the product’s stability under different storage conditions and the design of new and more efficient protocols of ANC extraction, purification, and concentration since they are the main substrates for the acylation reactions. Furthermore, new sources of ANCs could be studied, and engineered organisms with superior capacity to synthesize this type of compound could be developed. Although acylated ANCs exhibit promising features for their use as natural colorants, further studies are needed to evaluate their stability, in vitro and in vivo biological activities, and application in different products.

## Figures and Tables

**Table 1:** Source of anthocyanins, main anthocyanins, acyl donors, enzyme, acylated conjugates, and main findings of enzymatic acylation.

ANC Source	Main ANC	Acyl Donor	Enzyme	ANC Conjugate	Main Findings	Reference
Raspberry (*Rubus idaeus*)	Cyanidin-3-O-glucoside	Methyl salicylate	Novozym 435 (acrylic-resin-immobilized CALB)	Cyanidin-3-(6-salicyloyl) glucoside	Acylated ANCs showed improved thermal, photo-, and oxidative stabilities and also showed a good protective effect on oxidative stress damage	[37]
Red rose petals	Cyanidin-3,5-O-diglucoside	Fatty acids: caprylic acid, lauric acid, palmitic acid	Fermase CALBTM 10,000 (lipase B from *C. antarctica* immobilized on polyacrylate beads)	ANC lauric ester	The optimized reaction parameters were the following: acetonitrile (reaction medium), 40 °C (reaction temperature), 24 h (reaction time), 1:100 (molar ratio of reactants), 20 mg/mL (enzyme load), 100 mg/mL (molecular sieve load), 150 rpm (rate of shaking). ANC esters improved the thermo-oxidative stability of biscuit cream, also showed enhanced color stability in rice extrudate during thermal processing and storage	[38]
Pure C3G	Cyanidin-3-glucoside	Fatty acid methyl esters: methyl butyrate, methyl *n*-octanoate, methyl laurate, methyl myristate, methyl palmitate, and methyl stearate	Lipozyme 435 (recombinant lipase from *C. antarctica*)	Cyanidin-3-(6″-n-octanoyl)-glucoside, cyanidin-3-(6″-lauroyl)-glucoside, and cyanidin-3-(6″-myristoyl)-glucoside	C3G-n-octanoate had the highest thermostability and photostability. C3G-laurate had the highest cellular antioxidant capacity	[33]
Red wine	Malvidin 3-glucoside	Oleic acid (C18)	Lipase acrylic resin from *C. antarctica* (≥5000 U/g, recombinant, expressed in *Aspergillus* *niger*)	Mv3glc-C18	Preserved chromatic features (red-violet color) and antioxidant activity. Improved technological applications (potential in lipophilic systems, such as fats, oils, lipid-based food or cosmetic formulations)	[39]
Black rice (*Oryza sativa* L.)	Cyanidin-3-galactoside	Methyl benzoate, methyl salicylate, and methyl cinnamate	Novozym 435 (lipase B from *C. antarctica* immobilized on acrylic resin)	Cyanidin 3-(6″-benzoyl)-glucoside, cyanidin 3-(6″-salicyloyl)-glucoside, and cyanidin 3-(6″-cinnamoyl)-glucoside	Acylation with aromatic carboxylic acids enhanced thermostability and light-resistivity of ANCs. Cyanidin-3-(6″-cinnamoyl)-glucoside was the most stable	[40]
Red wine	Malvidin 3-glucoside	C4 to C16	Lipase acrylic resin from *C. antarctica* lipase B (≥5000 84 U/g, recombinant, expressed in *Aspergillus niger*)	Mv3glc-C4 to Mv3glc-C16	Increased lipophilicity. The maximum antioxidant activity was achieved when ANC was linked with caprylic acid (C8)	[41]
Blueberry (*Vaccinium corymbosum*)	Cyanidin-3-galactoside	Oleic acid and palmitic acid	Novozym 435 (*C. antarctica* lipase B 10,000 U/g)	Cyanidin-3-galactoside oleate and cyanidin-3-galactoside palmitate	Lipophilized ANC derivatives with free fatty acids improved oxidative stability under high temperature	[42]
Blackcurrant skin (*Ribes nigrum* L.)	Delphinidin-3-O-rutinoside, cyanidin-3-O-rutinoside, delphinidin-3-O-glucoside, cyanidin-3-O-glucoside	Octanoic acid (C8)	Lipase acrylic resin from *C. antarctica* lipase B (≥5000 U/g, recombinant, expressed in *Aspergillus niger*)	Dp3glc-C8 and Cy3glc-C8	Improved color stability (pH 3–7). Lower thermal degradation. Selective and preferential enzymatic acylation of cyanidin and delphinidin glucosides but not the corresponding rutinosides	[35]
Blackberry (*Rubus fruticosus* L.)	Cyanidin-3-O-glucoside	C4-C12	CalB immobilized in acrylic resin (≥5000 U/g, recombinant, expressed in *Aspergillus niger*)	Cy3glc-C4 to Cy3glc-C12	Improved color stability and lowered sensitivity to thermal degradation in an SDS micellar solution between pH 3 and 7	[2]
Alpine bearberry (*Arctostaphylos alpina*)	Cyanidin-3-O-galactoside	Lauric acid (C12)	*C. antarctica* lipase immobilized on acrylic resin (Novozyme 435) (≥5000 U/g, recombinant, expressed in *Aspergillus niger*)	Cyanidin-3-O-(6″-dodecanoyl) galactoside	Highest conversion yields (73%) obtained by acylation of cy-gal with lauric acid (C12). Improved lipophilicity and thermo-stability. Preserved UV-VIS absorbance and antioxidant properties	[43]
Blackberry (*Rubus fruticosus* L.)	Cyanidin-3-O-glucoside	Octanoic acid (C8)	Lipase B, powder form from *C. antarctica* (CalB) retained in composite membranes	Cy3glc-C8	Increased enzymatic activity of CalB-rich extract without enzyme purification. Improved yield of lipophilization reaction by 2.5-fold. Reusability of the membrane for three consecutive reaction cycles with the same ester conversion yield	[44]
Black rice (*Oryza sativa* L.)	Cyanidin-3-O-glucoside	Lauric acid (C12)	CalB, Novozym 435 (≥10,000 U/g, recombinant, expressed in *Aspergillus niger*)	Cy3glc-C12	Improved liposolubility, pH resistivity, and thermostability. Cy3glc-C12 promoted the proliferation of *Bifidobacteria* and *Lactobacillus* in the middle and later log phase and metabolization into phenolic acids	[34]
Blackcurrant (*Ribes nigrum*)	Delphinidin-3-O-glucoside, delphinidin-3-O-rutinoside, cyanidin-3-O-glucoside, and cyanidin-3-O-rutinoside	Lauric acid (C12)	Lipase acrylic resin from *C. antarctica* (≥5000 U/g, recombinant, expressed in *Aspergillus niger*)	Dp-glu-lauric acid, dp-rut-lauric acid, cy-glu-lauric acid, and cy-rut-lauric acid	Enhanced lipophilicity. Improved thermostability and capacity to inhibit lipid peroxidation	[45]
Tiliapo (*Sideroxylon palmeri*), trueno fruit (*Ligustrum japonicum*), bottlebrush flower (*Callistemon citrinus*), plum, and corn husks	Cyanidin-3-O-glucoside, cyanidin-3-O-rutinoside, cyanidin-3,5-diglucoside	Vinyl cinnamate, dihydrocinnamic acid, and cinnamic acid	Immobilized lipase from *C. antarctica* (≥5000 U/g, recombinant, expressed in *Aspergillus niger*)	Cy3-(4″- cinnamoyl) rutinoside, cy3,5-(6″-cinnamoyl) diglucoside, cy3-(6″-dihydrocinnamoyl) glucoside, cy3-(6″-dihydroferuloyl) glucoside, and cy3-(6″-dihydrosinapoyl) glucoside	Improved antioxidant activity and thermostability. Optimal reaction conditions involved tert-butanol as reaction media	[36]
Blueberry (*Vaccinium corymbosum*)	Cyanidin-3-O-glucoside	*p*-coumaric acid and caffeic acid	Lipase (Novozyme 435, ≥10,000 U/g, recombinant, expressed in *Aspergillus* *niger*)	ANCs acylated with *p*-coumaric acid (Co-An) and caffeic acid (Ca-An)	Stronger antioxidant activity and higher color stability during storage. *p*-coumaric and caffeic acids prevented ANCs from oxidation and breakdown	[46]
Hibiscus flower (*Hibiscus* *sabdariffa* L.)	Delphinidin 3-O-sambubioside	Octanoic acid (C8)	Lipase acrylic resin from *C. antarctica* lipase B (≥5000 U/g, recombinant, expressed in *Aspergillus niger*)	Dp3sam-C8	Stabilization of the quinoidal base (blue color) at neutral or moderate alkaline pH. Improved lipophilicity	[47]

Mv3glc, malvidin 3-glucoside; Dp3glc, delphinidin 3-O-glucoside; Dp3sam, delphinidin 3-O-sambubioside; Cy3glc, cyanidin 3-O-glucoside; Cy-gal, cyanidin 3-O-galactoside; Cy-glu, cyanidin 3-O-glucoside; Cy-rut, cyanidin 3-O-rutinoside; Dp-glu, delphinidin 3-O-glucoside; Dp-rut, delphinidin 3-O-rutinoside; CalB, *C. antarctica* lipase B.

**Table 2:** Similarities and differences between natural and synthetic acylation of anthocyanins.

Parameter or Characteristic	Natural Acylation	Synthetic Acylation
Site of acylation	Glycosidic residues	Glycosidic residues
Regioselectivity	6″-O, 4‴-O and 6‴-O depending on the species	6″-O (glucose and galactose) and 4″-O (rhamnose)
Enzyme	AATs	Lipase CalB from *Candida antarctica*. Just one study used a lipase from *Candida cylindrical* [34]
Acylating agents	Hydroxycinnamic acids (caffeic, *p*-coumaric, ferulic, sinapic), hydroxybenzoic acids (*p*-hydroxybenzoic and gallic), and aliphatic acids (acetic, malic, malonic, oxalic, succinic, tartaric, erucic, glutaric)	Methyl salicylate, methyl benzoate, methyl cinnamate, methyl butyrate, methyl laurate, methyl myristate, methyl palmitate, methyl stearate, n-octanoate, polyoxyethylene stearate, vinyl cinnamate, dihydrocinnamic acid, dihydroferuloyl acid, dihydrosinapic acid, cinnamic acid, *p*-coumaric acid, caffeic acid, butyric acid, hexanoic acid, octanoic acid, decanoic acid, lauric acid
Preferential acylation	Cinnamic acids	Aliphatic acids (lauric and octanoic acids). Long-chain fatty acids are better acyl donors than short-chain fatty acids
Polyacylation	Aromatic and aliphatic acylation may occur in the same molecule	Only one type of acylation is reported in enzymatic acylation studies
Enzyme immobilization	-	Lipase immobilization advantages: improved thermal and chemical stability, easy recycling and reuse, lower operating costs, and more prolonged enzyme survival
Sugars preferred	-	Mainly monosaccharide ANCs have easy access to the active site of the enzyme
Polarity	Decreases	Decreases
Water solubility	Decreases	Decreases
Oxidation	Changes in the ring orientation of ANC molecules influence the ease by which the hydrogen atoms from –OH groups are donated to free radicals, as well as the capacity of ANCs to support unpaired electrons	Acylation promotes a stronger resistance to H_2_O_2_ oxidation
Color stability	Increases	Increases
Resistance to pH increase	Higher	Higher
Photo-stability	Increases	Increases
Thermostability	Increases	Increases
References	[7,17,49]	[36,37,50]

Anthocyanin acyl-transferases (AATs), hydrogen peroxide (H_2_O_2_).

**Table 3:** Pharmacological effects of naturally acylated anthocyanins.

Major Acylated ANCs	Study Design	Main Findings	Reference
Cyanidin-3-caffeoylferuloylsophoroside-5-glucoside isolated from red cabbage	Streptozotocin-induced diabetic Wistar male rats, 130–150 g, *n* = 8. Three groups = Control, Diabetes, Diabetes + Red cabbage extract (RCE). Rats were given RCE daily (800 mg/kg) for 4 weeks	RCE lowered blood glucose and glycated hemoglobin concentrations, improved glucose tolerance, and raised serum insulin, proinsulin, and C-peptide levels. Increased the number of pancreatic β-cells in diabetic animals	[56]
Petunidin-3-O-rutinoside (*p*-coumaroyl)-5-O-glucoside isolated from black goji berry	SH-SY5Y cells. Cells were pre-protected with ANCs at concentrations of 50, 100, and 200 μg/mL for 12 h	Increased the autophagic flux, inhibited oxidative stress, and reduced inflammatory response and neuronal apoptosis with oxygen and glucose deprivation	[57]
Petunidin-coumaryl-rutinoside-glucoside isolated from purple potato	Zucker diabetic fatty rats (ZDF, fa/fa), 3 weeks old, *n* = 8. Rats were fed non-acylated ANC extract from bilberries (NAAB) or acylated ANC extract from purple potatoes (AAPP). Daily doses of 25 mg/kg (low dose) and 50 mg/kg (high dose) for 8 weeks	NAAB and AAPP improved lipid profiles. AAPP increased the glutamine/glutamate ratio and decreased levels of glycerol and improved insulin sensitivity, gluconeogenesis, and glycolysis. AAPP decreased the hepatic TBC1D1 and G6PC messenger RNA level, suggesting the regulation of gluconeogenesis and lipogenesis	[58]
Petunidin-coumaroyl-rutinoside-glucoside and peonidin-coumaroyl-rutinoside-glucoside isolated from purple potato	17 healthy subjects, 30 mL purple potato extract containing 152 mg of ANCs and 140 mg of other phenolics. Blood samples were taken in a range of 20–240 min	Suppressed postprandial plasma glucose and insulin peaks. Decreased plasma glucose and insulin at 20–60 min. Upregulation of postprandial level of insulin-like hormone FGF-19 after a high-carbohydrate meal	[59]
Diacylated ANCs cyanidin 3-dicaffeoyl sophoroside-5-glc and peonidin 3-dicaffeoyl sophoroside-5-glc isolated from purple sweet potato	Male Sprague–Dawley rats, 140–160 g, 6-weeks-old, *n* = 8. Three groups: normal control group (water), low-dose diacylated AF-PSP group (80 mg/kg), high-dose diacylated AF-PSP group (160 mg/kg). Blood samples collected from tail vein at 0, 15, 30, 60, 90, and 120 min	Low dose diacylated AF-PSP and high dose diacylated AF-PSP significantly decreased (*p* < 0.05 and *p* < 0.01, respectively) postprandial blood glucose levels after 30 min	[60]
Cya3SXylGlcGal, Cya3FXylGlcGal, and Cya3*p*CXylGlcGal isolated from black carrot	Colorectal adenocarcinoma (HT-29) and promyelocytic leukemia (HL-60) cells. BC-ARE concentrations: 0.0–2.0 mg/mL for 24 h	BC-ARE at 2.0 mg/mL suppressed about 80% of the growth of HT-29 and HL-60 cells	[61]
C3G-Mal, Pr3G-Mal, and P3G-Mal isolated from purple maize	Inhibitory effect on α-amylase and dipeptidyl peptidase-4 (DPP-IV). PMW concentrations: 0.05–1.0 mg/mL	PMW inhibited α-amylase with an IC_50_ from 109.5 to 172.7 μg/mL. PMW repressed DPP-IV activity with an IC_50_ from 65.5 to 702.7 μg/mL	[55]
Cyanidin succinyl glucoside and cyanidin malonyl glucoside isolated from purple highland barley	PC12 cells were exposed to CoCl_2_ for 12 h to mimic hypoxic conditions and treated with various concentrations of PAE (25–400 μg/mL)	PAE at 400 μg/mL showed the highest protective effect on PC12 cells against the hypoxic treatment, retaining 76.1% of the cell viability	[62]

RCE, red cabbage extract; BC-ARE, black carrot anthocyanin-rich extract; PMW, purple maize anthocyanin-rich water extract; PAE, purified anthocyanin extract from purple highland barley bran; NAAB, non-acylated ANC extract from bilberries; AAPP, acylated ANC extract from purple potatoes; AF-PSP, ANC fraction from purple sweet potato; ZDF fa/fa, Zucker diabetic fatty rats; Cya3SXylGlcGal, cyanidin 3-sinapoylxylosylglucosylgalactoside; Cya3FXylGlcGal, cyanidin 3-feruloylxylosylglucosylgalactoside; Cya3pCXylGlcGal, cyanidin 3-p-coumaroylxylosylglucosylgalactoside; C3G-Mal, cyanidin-3-(6′-malonylglucoside); Pr3G-Mal, pelargonidin-3-(6′-malonylglucoside); P3G-Mal, peonidin-3-(6′-malonylglucoside).

## Data Availability

No new data were created or analyzed in this study. Data sharing is not applicable to this article.
